# Probiotic reduces bacterial translocation in type 2 diabetes mellitus: A randomised controlled study

**DOI:** 10.1038/s41598-017-12535-9

**Published:** 2017-09-21

**Authors:** Junko Sato, Akio Kanazawa, Kosuke Azuma, Fuki Ikeda, Hiromasa Goto, Koji Komiya, Rei Kanno, Yoshifumi Tamura, Takashi Asahara, Takuya Takahashi, Koji Nomoto, Yuichiro Yamashiro, Hirotaka Watada

**Affiliations:** 1 Department of Metabolism & Endocrinology, Juntendo University Graduate School of Medicine, Tokyo, 113-8421 Japan; 2 Center for Therapeutic Innovations in Diabetes, Juntendo University Graduate School of Medicine, Tokyo, 113-8421 Japan; 3 Center for Identification of Diabetic Therapeutic Targets, Juntendo University Graduate School of Medicine, Tokyo, 113-8421 Japan; 4 Sportology Center, Juntendo University Graduate School of Medicine, Tokyo, 113-8421 Japan; 5 Probiotics Research Laboratory, Juntendo University Graduate School of Medicine, Tokyo, 113-8421 Japan; 6 Yakult Central Institute, Tokyo, 186-8650 Japan

## Abstract

Gut bacterial translocation to the blood may play an important role in the development of insulin resistance in type 2 diabetes. Here, we performed an interventional randomised control study to investigate whether probiotics could reduce bacterial translocation and cause changes in the gut microbiota. Seventy Japanese patients with type 2 diabetes were randomised to two groups: the probiotic group drank *Lactobacillus casei* strain Shirota-fermented milk, while the control group ingested no probiotics. The trial was conducted for 16 weeks. At baseline, 8 and 16 weeks, the gut microbiota composition in feces and blood, fecal organic acids, and other biochemical parameters were measured. At the end of the study, the fecal counts of the *Clostridium coccoides* group and *Clostridium leptum* subgroup in the probiotic group were significantly higher than in the control group. As expected, the fecal counts of total *Lactobacillus* were significantly higher in the probiotic group. Intriguingly, the total count of blood bacteria was significantly lower in the probiotic group. However, fecal organic acids were comparable between the two groups. Our results showed that probiotic administration reduced bacterial translocation and altered the gut microbiota in Japanese patients with type 2 diabetes mellitus.

## Introduction

Over the past decades, the incidence of diabetes has increased worldwide^1^. A great change in dietary habits characterized by an increased intake of fat is considered to be responsible for the dramatic rise in metabolic diseases^2^. In this situation, the gut microbiota is a great topic in the research of this field.

Short-chain fatty acids formed from the fermentation of dietary fiber by the gut microbiota have been found to be associated with incretin secretion^3^, intestinal gluconeogenesis^4^, insulin sensitivity in adipocytes^5^, and insulin secretion via activation of the parasympathetic nerve^6^. Thus, interesting roles of the gut microbiota in glucose metabolism are closely linked to the pathogenesis of diabetes and manipulation of the human gut microbiota might provide important clues regarding a new therapeutic target for diabetes.

The leaky gut has also been attracting a great deal of attention in the context of metabolic diseases. In particular, a fat-rich diet was shown to lead to changes in the gut microbiota that strongly increased intestinal permeability due to malfunction of tight junction proteins such as occuludin and ZO-1^7^. This results in increased plasma levels of lipopolysaccharide (LPS), a condition known as “metabolic endotoxemia”, which causes low-grade inflammation and eventually insulin resistance^8^. In animal models, our group previously showed that probiotic administration improved insulin resistance and glucose intolerance in diet-induced obesity mice with reduction in endotoxemia^9^, and an epidemiological study also determined that an increased blood concentration of 16 s ribosomal DNA from gut bacteria was a risk factor for developing diabetes in a general population^10^. Considering the results of these previous studies, bacterial translocation due to leaky gut could play a still unidentified role in the pathophysiology of diabetes through insulin resistance and/or other unknown mechanisms.

Previously, we reported for the first time the presence of gut dysbiosis and a higher rate of detection of live gut bacteria in the blood of Japanese patients with type 2 diabetes compared to non-diabetes^11^, suggesting that bacterial translocation occurs in type 2 diabetes. However, the causal relation between gut dysbiosis and bacterial translocation remained unclear because our previous study used a cross-sectional design. Addressing this issue would require an interventional trial using probiotics that can modify the gut microbiota in type 2 diabetes mellitus. Among various probiotics, the beneficial effect of the probiotic *Lactobacillus casei* strain Shirota (LcS) on the gut microbiota and intestinal environment has been proven in healthy individuals^12^ and the elderly^13^. In an animal diabetes model, improvement of glucose metabolism by LcS was reported previously^14^. Furthermore, fecal butyric acids were known to play protective roles in intestinal barrier function through reassembly of tight junctions^15^, and other fecal organic acids also improved intestinal barrier function *in vitro* ^16^ and *in vivo* model^17^.

Therefore, we performed an interventional randomised control study to investigate the effects of daily intake of probiotic LcS-fermented milk on the gut microbiota, fecal organic acids, and bacterial translocation in Japanese patients with type 2 diabetes mellitus.

## Results

### Baseline characteristics

**Figure 1:**
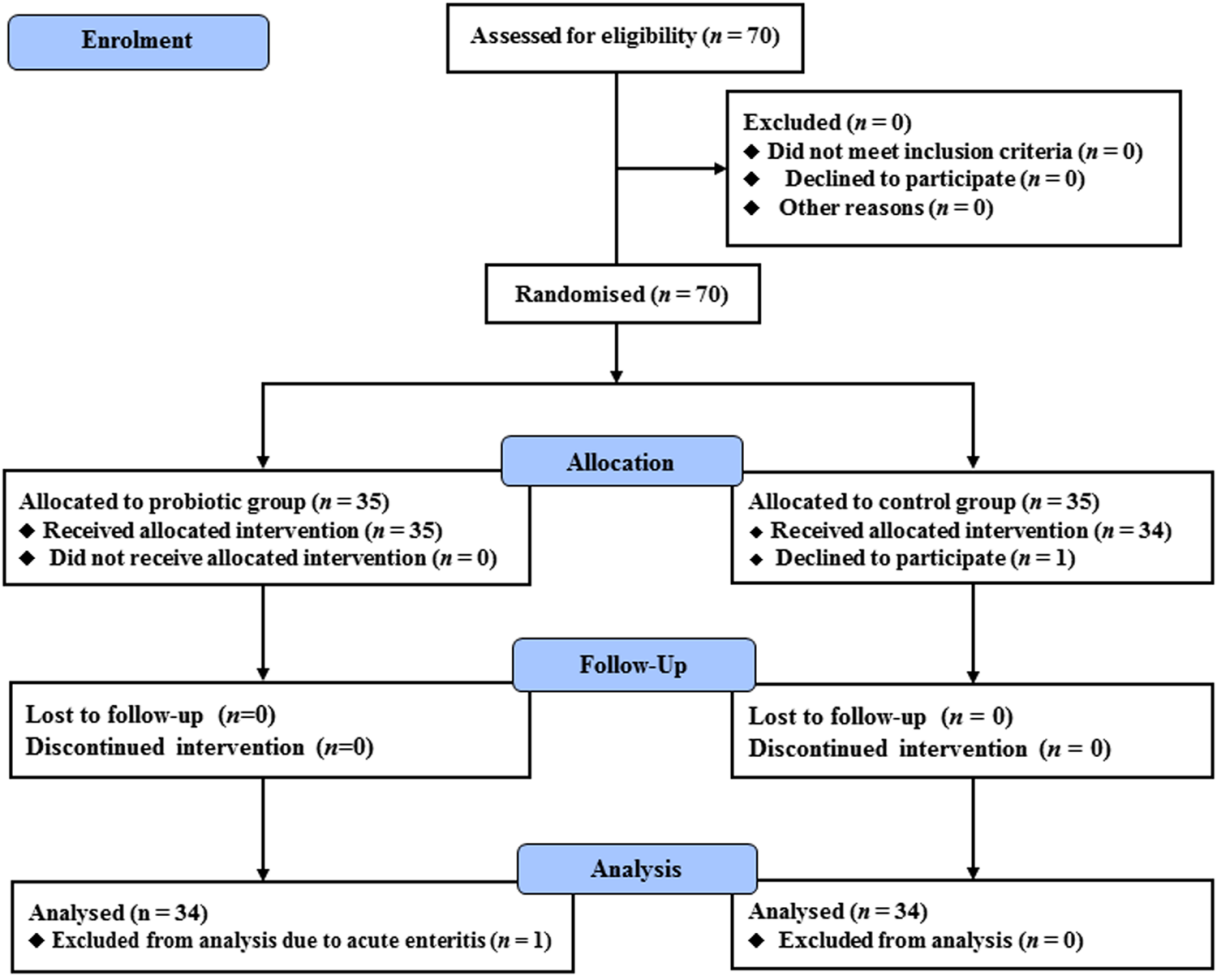
Flow diagram of patient recruitment. Seventy patients were randomly allocated to either the probiotic group or the control group. One patient in the control group declined to participate in the study, and one patient in the probiotic group was excluded from the analysis due to the onset of acute enteritis during the study period. The remaining 68 patients were analysed.

Of the 70 patients recruited in this study, 35 were assigned to the probiotic group and 35 to the control group. In the probiotic group, 35 patients completed the 16-week intervention but one patient was excluded from the final analysis due to onset of acute enteritis. In the control group, 34 patients completed the 16-week trial, while one declined participation after randomisation (Fig. 1). The baseline characteristics of the patients who completed the study are summarised in Table 1. The rate of men in the probiotic group was significantly higher than the control group (*p* < 0.05). However, other parameters including age, body mass index (BMI), fasting blood glucose and HbA1c were comparable between the two groups (Table 1). Therefore, the effect of difference in rate of sex on the results was considered to be small.

**Table 1:** Characteristics of study subjects at baseline.

	Control (*n* = 34)	Probiotic (*n* = 34)
Sex (male/female)	20/14	29/5
Age (years)	65.0 ± 8.3	64.0 ± 9.2
BMI (kg/m^2^)	24.6 ± 2.6	24.2 ± 2.6
Duration of diabetes (years)	12.2 ± 7.2	13.9 ± 9.6
HbA1c (%)	6.8 ± 0.5	6.9 ± 0.5
Glycoalbumin (%)	17.6 ± 3.4	17.6 ± 2.3
Fasting blood glucose (mg/dL)	129.5 ± 26.6	128.5 ± 19.1
Fasting C-peptide (ng/ml)	1.8 ± 0.7	1.7 ± 0.6
T-CHO (mg/dL)	182.9 ± 28.2	188.2 ± 31.7
HDL-C (mg/dL)	55.7 ± 14.7	54.0 ± 15.2
TG (mg/dL)	109.4 ± 45.0	104.7 ± 35.6
hs-CRP (mg/dL)	403.5 (166.0–757.0)	370.5 (218.0–610.0)
TNF-α (pg/mL)	0.9 ± 0.3	1.1 ± 0.5
IL-6 (pg/mL)	1.8 ± 0.9	1.7 ± 1.2
Adiponectin (μg/mL)	8.3 ± 2.9	10.3 ± 7.2
LBP (μg/mL)	11.2 ± 3.9	10.0 ± 2.2
Medication for diabetes		
No medication	8	6
Insulin only or with oral therapy	4	3
Oral therapy only		
SU	7	9
Metformin	16	17
Thiazolidine	1	5
DPP-4 inhibitor	16	22
Glinide	6	3
SGLT2 inhibitor	1	2
GLP-1-receptor agonist	1	0

Data are mean ± SD or median (interquartile range; 25–75%). BMI, Body mass index; T-CHO, total cholesterol; HDL-C, high-density lipoprotein cholesterol; TG, triglycerides; hs-CRP, high-sensitivity C-reactive protein; IL-6, interleukin-6; TNF-α, tumor necrosis factor-α; LBP, lipopolysaccharide binding protein; SU, sulfonylurea; DPP-4 inhibitor, dipeptidyl peptidase-4 inhibitor; SGLT2 inhibitor, sodium-dependent glucose cotransporter-2 inhibitor; GLP-1-receptor agonist, glucagon-like peptide-1-receptor agonist.

### Serial changes of fecal microbiota before and after probiotic administration

Table 2 shows the serial changes of fecal microbiota after probiotic administration. At baseline there were no significant differences in fecal bacterial counts between the two groups. However, the counts of total *Lactobacillus* and the *L. casei* subgroup at 8 and 16 weeks were significantly higher in the probiotic group compared with the control group (*p* < 0.01) and were significantly increased compared with baseline (*p* < 0.01). The detection rate of the *L. casei* subgroup in feces was significantly higher in the probiotic group compared with the control group at 8 and 16 weeks (*p* < 0.01). In addition, the counts of the *L. gasseri* subgroup at 8 and 16 weeks (*p* < 0.05) and the *L. reuteri* subgroup at 16 weeks (*p* < 0.05) were significantly increased in the probiotic group compared with baseline, while no such changes were observed in the control group. On the other hand, the counts of *Bifidobacterium, Atopobium* cluster, total *Lactobacillus, and L. fermentum* at 16 weeks were significantly increased and those of *Prevotella* at 16 weeks were significantly decreased in the control group compared with baseline (*p* < 0.05). Further, among the obligate anaerobes, the counts of the *C. coccoides* group and the *C. leptum* subgroup at 16 weeks after probiotic administration were significantly higher in the probiotic group compared with the control group (*p* < 0.05), and linear mixed model analysis showed the same results (S2 Table).

**Table 2:** Fecal microbiota at 0, 8 and 16 weeks in diabetic patients with and without probiotic administration.

	0 weeks				8 weeks				16 weeks			
	Control		Probiotic		Control		Probiotic		Control		Probiotic	
Total bacteria	10.2 ± 0.6	(100.0)	10.3 ± 0.6	(100.0)	10.2 ± 0.5	(100.0)	10.3 ± 0.5	(100.0)	10.2 ± 0.6	(100.0)	10.4 ± 0.4	(100.0)
Obligate anaerobes												
*C. coccoides* group	9.4 ± 0.7	(100.0)	9.5 ± 0.8	(100.0)	9.3 ± 0.6	(100.0)	9.6 ± 0.5	(100.0)	9.5 ± 0.6	(97.1)	9.8 ± 0.4*	(100.0)
*C. leptum* subgroup	9.5 ± 0.8	(100.0)	9.6 ± 0.7	(100.0)	9.4 ± 0.8	(100.0)	9.5 ± 0.7	(100.0)	9.4 ± 0.8	(100.0)	9.8 ± 0.5*	(100.0)
*Bacteroides fragilis* group	8.7 ± 0.7	(100.0)	8.8 ± 0.8	(100.0)	8.6 ± 0.6	(94.1)	8.6 ± 0.7	(100.0)	8.6 ± 0.6	(97.1)	8.8 ± 0.7	(100.0)
*Bifidobacterium*	8.7 ± 1.0	(97.1)	8.9 ± 1.3	(100.0)	8.9 ± 1.1	(97.1)	8.8 ± 1.2	(100.0)	9.0 ± 1.1^$^	(100.0)	9.0 ± 1.0	(97.1)
*Atopobium* cluster	9.2 ± 0.9	(100.0)	9.3 ± 0.6	(100.0)	9.3 ± 0.8	(97.1)	9.4 ± 0.5	(100.0)	9.4 ± 0.8^$^	(97.1)	9.5 ± 0.6	(100.0)
*Prevotella*	8.7 ± 1.2	(47.1)	7.7 ± 1.5	(64.7)	8.2 ± 1.7	(55.9)	8.2 ± 1.4	(61.8)	8.3 ± 1.6^$^	(64.7)	8.0 ± 1.6	(70.6)
*Akkermansia muciniphila*	7.4 ± 1.1	(64.7)	7.4 ± 1.6	(64.7)	7.6 ± 1.4	(61.8)	7.3 ± 1.5	(64.7)	7.5 ± 1.2	(67.6)	7.9 ± 1.6	(61.8)
*C. difficle*	4.7 ± 1.8	(8.8)	<2.3	(0.0)	3.9 ± 1.0	(11.8)	4.3 ± 1.3	(11.8)	3.6 ± 0.6	(17.6)	3.7 ± 1.2	(14.7)
*C. perfringens*	4.8 ± 1.2	(26.5)	5.2 ± 1.8	(35.3)	4.7 ± 2.0	(32.4)	5.4 ± 1.8	(26.5)	4.6 ± 1.4	(41.2)	4.6 ± 1.1	(26.5)
Facultative anaerobes												
Total *Lactobacillus*	6.2 ± 1.5	(100.0)	5.7 ± 1.2	(100.0)	6.2 ± 1.6	(100.0)	7.6 ± 0.6**^$$^	(100.0)	6.7 ± 1.4^$^	(100.0)	7.7 ± 0.6**^$$^	(100.0)
*L. gasseri* subgroup	5.8 ± 1.6	(76.5)	5.1 ± 1.2	(85.3)	5.6 ± 1.7	(85.3)	5.5 ± 1.1^$^	(82.4)	5.8 ± 1.4	(88.2)	5.6 ± 1.1^$^	(79.4)
*L. brevis*	4.8 ± 1.5	(38.2)	4.0 ± 0.6	(23.5)	3.8 ± 0.9	(29.4)	3.7 ± 1.0	(38.2)	4.3 ± 1.4	(38.2)	4.2 ± 1.1	(29.4)
*L. casei* subgroup	5.0 ± 1.2	(41.2)	4.9 ± 1.1	(29.4)	4.6 ± 0.7	(38.2)	7.4 ± 0.7** ^$$^	(100.0)**	5.0 ± 1.3	(38.2)	7.4 ± 0.8**^$$^	(100.0)**
*L. fermentum*	5.9 ± 1.1	(29.4)	6.0 ± 1.2	(35.3)	6.4 ± 1.3	(32.4)	5.6 ± 1.1	(38.2)	6.7 ± 1.1^$^	(41.2)	6.1 ± 1.1	(38.2)
*L. fructivorans*	4.5 ± 1.0	(14.7)	3.3 ± 1.3	(8.8)	3.3 ± 0.3	(5.9)	3.5 ± 0.8	(8.8)	<2.3	(0.0)	4.9	(2.9)
*L. plantarum* subgroup	4.7 ± 1.6	(76.5)	4.4 ± 1.0	(88.2)	4.2 ± 1.2	(79.4)	4.3 ± 1.0	(85.3)	4.5 ± 1.3	(70.6)	4.3 ± 1.0	(85.3)
*L. reuteri* subgroup	5.2 ± 1.5	(61.8)	4.8 ± 1.0	(76.5)	5.5 ± 1.3	(67.6)	4.9 ± 1.2	(79.4)	5.5 ± 1.5	(70.6)	5.3 ± 1.0^$^	(76.5)
*L. ruminis* subgroup	4.9 ± 1.6	(61.8)	5.0 ± 1.7	(50.0)	5.7 ± 1.8	(52.9)	5.3 ± 1.5	(61.8)	5.1 ± 1.9	(61.8)	4.9 ± 2.0	(67.6)
*L. sakei* subgroup	4.6 ± 1.7	(38.2)	4.2 ± 0.8	(23.5)	4.8 ± 1.3	(44.1)	5.1 ± 1.2	(32.4)	5.4 ± 1.7	(47.1)	4.5 ± 1.8	(29.4)
*Enterobacteriaceae*	7.1 ± 1.3	(91.2)	7.1 ± 1.2	(97.1)	6.9 ± 0.9^$^	(85.3)	7.0 ± 1.2	(91.2	6.9 ± 1.0	(73.5)	7.0 ± 0.9	(85.3)
*Enterococcus*	6.3 ± 1.3	(88.2)	6.0 ± 1.3	(85.3)	6.2 ± 1.0	(82.4)	6.2 ± 1.2	(82.4)	6.0 ± 1.3	(82.4)	6.5 ± 1.2^$^	(76.5)
*Streptococcus*	8.4 ± 1.1	(100.0)	8.5 ± 0.8	(100.0)	8.4 ± 0.9	(97.1)	8.3 ± 0.6	(100.0)	8.4 ± 1.3	(97.1)	8.5 ± 0.8	(100.0)
*Staphylococcus*	4.7 ± 1.0	(85.3)	4.7 ± 0.8	(91.2)	4.4 ± 0.8^$^	(91.2)	4.4 ± 0.8	(94.1)	4.5 ± 0.9	(73.5)	4.4 ± 0.7	(91.2)
Aerobes												
*Pseudomonas*	4.7 ± 1.9	(17.6)	3.7 ± 0.8	(23.5)	4.2 ± 0.8	(23.5)	4.5 ± 0.7	(23.5)	3.9 ± 1.0	(26.5)	4.6 ± 0.7	(26.5)
Administration of *L. casei* strain Shirota	<5.0	(0.0)	5.8	(2.9)	<5.0	(0.0)	7.3 ± 0.8	(97.1)**	6.4	(2.9)	7.2 ± 0.8	(91.2)**

^$^ *p* < 0.05 vs. baseline, ^$$^ *p* < 0.01 vs. baseline, *p < 0.05 vs. Control, **p < 0.01 vs. Control.

The results are expressed as mean ± SD (log_10_ cells/g of feces). Detection rate (%).

### Serial changes of fecal organic acids and pH before and after probiotic administration

The results of serial changes of fecal organic acids and pH are presented in Table 3. At baseline, there were no significant differences in the fecal concentrations of total organic acids between the two groups, but the concentration of butyric acid was significantly higher in the probiotic group compared with the control group (*p* < 0.05). At 8 and 16 weeks, the concentrations of total organic acids and butyric acid were significantly decreased in the probiotic group compared with baseline (*P* < 0.05), and fecal pH at 8 and 16 weeks in the probiotic group was significantly increased compared with baseline. However, the levels of these organic acids and the fecal pH at 8 and 16 weeks were not significantly different between the two groups, and linear mixed model analysis (S3 Table) showed the significant decrease of valeric acids at 16 weeks in the probiotic group compared to the control group (*p* < 0.05).

**Table 3:** Fecal organic acids and pH at 0, 8 and 16 weeks in diabetic patients with and without probiotic administration.

	0 weeks				8 weeks				16 weeks			
	Control		Probiotic		Control		Probiotic		Control		Probiotic	
Total organic acids	96.1 ± 42.7	(100.0)	112.7 ± 37.3	(100.0)	91.2 ± 36.5	(100.0)	98.2 ± 36.8^$^	(100.0)	107.1 ± 47.5	(100.0)	102.2 ± 33.7^$^	(100.0)
Acetic acid	57.4 ± 26.9	(100.0)	66.2 ± 23.7	(100.0)	52.9 ± 22.0	(100.0)	58.2 ± 22.3	(100.0)	59.2 ± 24.8	(100.0)	57.0 ± 21.6^$^	(100.0)
Propionic acid	21.0 ± 11.6	(100.0)	23.1 ± 9.4	(100.0)	19.4 ± 9.4	(100.0)	21.0 ± 10.3	(100.0)	23.6 ± 13.2	(100.0)	21.9 ± 9.1	(100.0)
Butyric acid	12.6 ± 7.6	(85.3)	16.7 ± 9.2*	(100.0)	12.2 ± 7.6	(91.2)	12.5 ± 8.2^$$^	(97.1)	14.9 ± 10.4	(100.0)	14.2 ± 9.0^$^	(100.0)
Isovaleric acid	4.1 ± 3.6	(55.9)	3.3 ± 1.9	(70.6)	3.5 ± 2.9	(73.5)	3.3 ± 1.8	(67.6)	4.8 ± 3.5	(70.6)	3.5 ± 2.1	(70.6)
Valeric acid	3.3 ± 2.3	(50.0)	2.9 ± 1.2	(64.7)	2.8 ± 1.7	(67.6)	2.1 ± 1.1^$$^	(76.5)	4.3 ± 3.8	(70.6)	2.9 ± 1.5	(70.6)
Succinic acid	2.9 ± 6.6	(61.8)	2.4 ± 4.2	(70.6)	2.8 ± 5.5	(67.6)	3.4 ± 10.0^$$^	(61.8)	2.9 ± 5.1	(50.0)	4.8 ± 14.7	(76.5)*
Formic acid	1.6 ± 1.5	(64.7)	1.1 ± 0.6	(73.5)	1.6 ± 2.2	(76.5)	1.2 ± 0.9	(79.4)	1.8 ± 2.2	(76.5)	1.0 ± 0.6	(73.5)
Lactic acid	1.5 ± 0.8	(14.7)	4.2	(2.9)	1.1 ± 0.8	(11.8)	0.6 ± 1.1	(8.8)	1.2 ± 0.8	(17.6)	0.9 ± 0.5	(20.6)
pH	6.5 ± 0.6	(100.0)	6.4 ± 0.6	(100.0)	6.7 ± 0.6	(100.0)	6.7 ± 0.6^$$^	(100.0)	6.6 ± 0.5	(100.0)	6.7 ± 0.5^$$^	(100.0)

^$^ *p* < 0.05 vs. baseline, ^$$^ *p* < 0.01 vs. baseline, *p < 0.05 vs. Control,

Fecal organic acids are expressed as the mean ± SD (µmol/g of feces).

Detection rate (%).

### Serial changes of counts and detection rates of gut bacteria in the blood before and after probiotic administration

**Figure 2:**
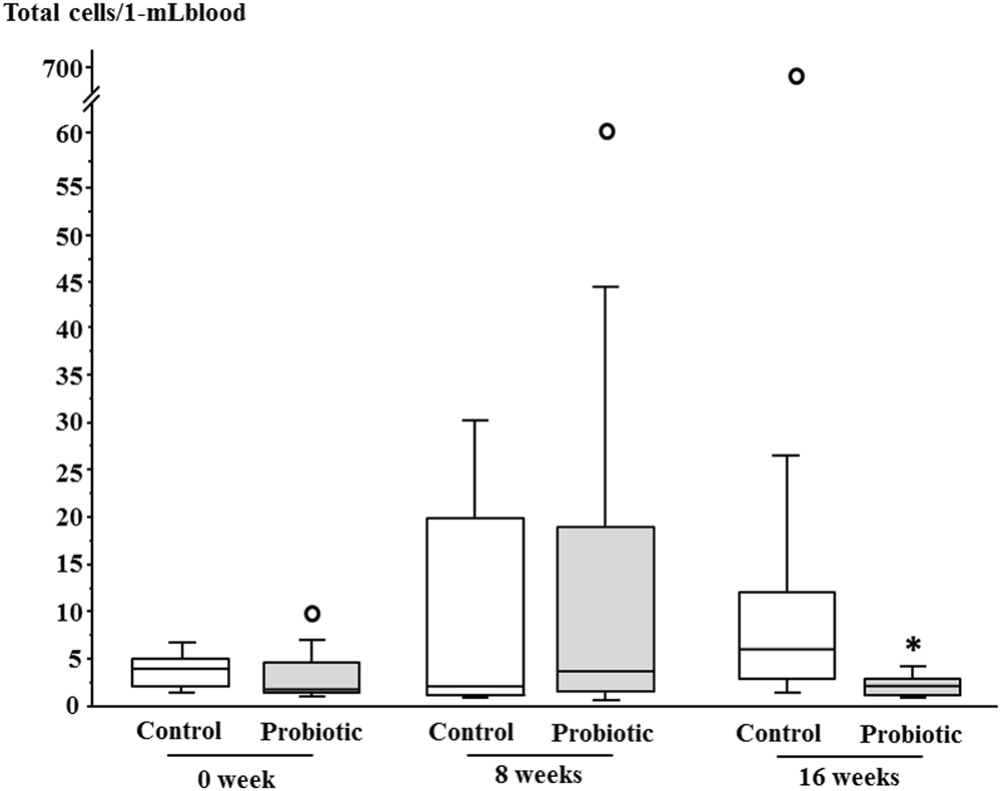
Total counts of bacteria in blood of the control and probiotic groups during the study period. Horizontal bars represent medians, and columns indicate interquartile ranges (IQRs). When a data point was above Q3 (the third quartile) + 1.5 × IQR or below Q1 (the first quartile) − 1.5 × IQR, it was defined as an outlier (white circle). Upper or lower whisker represents the maximum or minimum value if there are no outliers. Upper whiskers at 0 and 8 weeks in the probiotic group and 16 weeks in the control group represented the maximum values within Q3 + 1.5 × IQR as outliers were found. On the other hand, all lower whiskers represented the minimum values. *p < 0.05 vs. Control

Gut bacteria in the blood of diabetes patients were detected in both the control and the probiotic groups during the study period; the detection rates were comparable between the two groups (Table 4), and did not change significantly between baseline and 16 weeks. However, the total count of gut bacteria in the blood at 16 weeks was significantly lower in the probiotic group compared with the control group (*p* < 0.05). Figure 2 presented the results of total counts of gut bacteria in blood as graphs.

**Table 4:** Counts and detection rates of gut bacteria in the blood of diabetes mellitus patients with and without probiotic administration.

	0 weeks				8 weeks				16 weeks			
	Control		Probiotic		Control		Probiotic		Control		Probiotic	
Total bacteria	4.5 (2.3–5.3)	(20.6)	2.4 (2.1–4.1)	(14.7)	2.3 (1.6–20.2)	(20.6)	4.4 (2.1–19.0)	(14.7)	6.0 (3.3–12.4)	(29.4)	1.8 (1.0–3.2)*	(20.6)
Obligate anaerobe												
*C. coccoides* group	3.0	(2.9)	1.0	(2.9)	2.9 (1.5–4.2)	(5.9)	9.9	(2.9)	1.8 (1.4–2.1)	(8.8)	1.6 (1.3–1.9)	(5.9)
*C. leptum* subgroup	2.1 (1.5–2.5)	(11.8)	2.1 (1.5–2.4)	(8.8)	2.6 (2.1–6.0)	(11.8)	2.8 (1.1–10.1)	(8.8)	3.8 (3.2–4.4)	(17.6)	1.8 (1.8–2.7)	(8.8)
*Atopobium* cluster	4.0 (2.3–5.7)	(5.9)	5.1 (1.4–8.7)	(5.9)	7.0 (1.6–16.3)	(8.8)	37.1	(2.9)	4.3	(2.9)	1.6	(2.9)
*Bacteroides fragilis* group	ND	(0.0)	ND	(0.0)	ND	(0.0)	1.2	(2.9)	ND	(0.0)	ND	(0.0)
*Prevotella*	ND	(0.0)	ND	(0.0)	ND	(0.0)	19	(2.9)	8.4 (6.4–10.4)	(5.9)	ND	(0.0)
*Bifidobacterium*	ND	(0.0)	ND	(0.0)	ND	(0.0)	ND	(0.0)	ND	(0.0)	ND	(0.0)
*C. perfringens*	ND	(0.0)	ND	(0.0)	ND	(0.0)	ND	(0.0)	ND	(0.0)	ND	(0.0)
Facultative anaerobes												
*Streptococcus*	1.7 (1.0–4.5)	(8.8)	2.6	(2.9)	20.2	(2.9)	1.3 (1.0–2.1)	(11.8)	2.2 (1.3–3.3)	(17.6)	1.0 (1.0–1.2)	(8.8)
*Staphylococcus*	2.7	(2.9)	ND	(0.0)	ND	(0.0)	ND	(0.0)	ND	(0.0)	ND	(0.0)
*Enterobacteriaceae*	ND	(0.0)	ND	(0.0)	ND	(0.0)	ND	(0.0)	ND	(0.0)	ND	(0.0)
*Enterococcus*	ND	(0.0)	ND	(0.0)	ND	(0.0)	ND	(0.0)	ND	(0.0)	ND	(0.0)
Total *Lactobacillus*	ND	(0.0)	ND	(0.0)	ND	(0.0)	ND	(0.0)	ND	(0.0)	ND	(0.0)
Aerobes												
*Pseudomonas*	ND	(0.0)	ND	(0.0)	ND	(0.0)	ND	(0.0)	ND	(0.0)	ND	(0.0)

Data are median (interquartile range; 25–75%) (cells per 1-mL blood sample).

Detection rate (%), ND: Not detected

*p < 0.05 vs. Control.

### Serial changes of clinical parameters and inflammatory markers before and after probiotic administration

As shown by Table 5, the level of hs-CRP at 16 weeks was significantly increased in the probiotic group compared with baseline (*p* < 0.01), however changes in hs-CRP were not significantly different between the two groups. Then, changes in other inflammatory markers such as IL-6, TNF-α, and LBP were comparable between the two groups. The levels of HbA1c at 16 weeks in the control (*p* < 0.05) and probiotic (*p* < 0.01) groups were slightly increased compared with baseline, however changes of HbA1c, fasting blood glucose, and glycoalbumin were comparable between the two groups. The levels of T-CHO in the probiotic group and HDL-C in the control group were significantly increased compared with baseline (*p* < 0.05). Additionally, linear model analysis (S4 Table) showed the significant decrease of HDL-C at 8 weeks in the probiotic group compared to the control group (*p* < 0.05).

**Table 5:** Changes of clinical parameters in diabetes mellitus patients with and without probiotic administration.

	Control (n = 34)			Probiotic (n = 34)		
	8 weeks	16 weeks	Change	8 weeks	16 weeks	Change
BMI (kg/m^2^)	23.9 ± 2.6	24.0 ± 2.6	0.0 ± 0.9	24.2 ± 2.5	24.2 ± 2.6	0.0 ± 0.7
HbA1c (%)	6.9 ± 0.5	6.9 ± 0.5	0.1 ± 0.4^$^	6.9 ± 0.6	7.1 ± 0.7	0.2 ± 0.3^$$^
Glycoalbumin (%)	17.6 ± 3.2	17.6 ± 3.3	−0.1 ± 1.5	17.7 ± 2.4	17.9 ± 2.8	0.3 ± 1.4
Fasting blood glucose (mg/dL)	130.2 ± 30.0	137.1 ± 31.7	7.6 ± 20.8	131.7 ± 24.2	133.1 ± 21.6	4.6 ± 16.6
Fasting C-peptide (ng/ml)	1.9 ± 0.7	1.9 ± 0.7	0.0 ± 0.5	1.8 ± 0.6^$^	1.8 ± 0.8	0.1 ± 0.4
T-CHO (mg/dL)	188.2 ± 31.2	188.5 ± 28.6	6.3 ± 17.9	193.8 ± 33.5^$^	191.9 ± 26.5^$^	3.7 ± 14.3^$^
HDL-C (mg/dL)	57.6 ± 14.5^$^	56.5 ± 14.6^$^	1.0 ± 8.4^$^	53.8 ± 14.7	54.1 ± 13.9	0.0 ± 5.9
TG (mg/dL)	115.9 ± 68.2	122.1 ± 104.5	14.0 ± 91.3	117.5 ± 58.7	106.4 ± 40.8	1.7 ± 25.2
hs-CRP (mg/dL)	441.0 (243.0–668.0)	453.5 (293.0–952.0)	32.5 (−110.0–317.0)	435.0 (274.0–768.0)	423.5 (277.0–865.0)^$$^	92.0 (−2.0–286.0)^$$^
TNF-α (pg/mL)	0.9 ± 0.2	0.9 ± 0.3	0.0 ± 0.3	1.1 ± 0.5*	1.1 ± 0.5	0.0 ± 0.5
IL-6 (pg/mL)	1.9 ± 1.3	2.0 ± 0.9	0.2 ± 1.0	1.8 ± 1.1	2.1 ± 2.6	0.4 ± 2.8
Adiponectin (μg/mL)	8.1 ± 2.7	8.2 ± 2.9	−0.1 ± 1.1	10.3 ± 6.6	10.3 ± 6.9	0.0 ± 3.5
LBP (μg/mL)	10.7 ± 3.7	10.7 ± 3.7	−0.5 ± 4.0	11.1 ± 4.7	10.0 ± 3.2	0.0 ± 3.8

See Table 1 for abbreviations. Data are mean ± SD or median (interquartile range; 25–75%).

The change is expressed as the value measured at 16 weeks minus baseline value (0 weeks).

^$^ *p* < 0.05 vs. baseline, ^$$^ *p* < 0.01 vs. baseline, *p < 0.05 vs. Control.

## Discussion

There have been several reports thus far regarding the effects of probiotic LcS on the gut microbiota and inflammatory markers in metabolic syndrome^18,19^ and on insulin sensitivity in healthy individuals^20^. In addition, the effects of other probiotics on insulin sensitivity^21^ and inflammatory markers^22^ in diabetes have been reported. However, no clinical trials have adopted a primary outcome of bacterial translocation specifically in patients with type 2 diabetes mellitus. In the present study, we demonstrated for the first time that administration of LcS-fermented milk could reduce gut bacterial translocation to the blood in type 2 diabetes mellitus.

Previous studies clearly showed that persons with higher levels of gut bacterial DNA in the blood were at risk of developing diabetes in the future^10^, and in animal models a high-fat diet caused gut bacterial DNA to be phagocytosed by macrophages and to subsequently accumulate in the adipose tissue^23^. Furthermore, the present study confirmed the presence of bacteremia in type 2 diabetes, as did our previous study^11^. Thus, gut bacterial translocation to the blood might play important roles in chronic low-grade inflammation in obesity and diabetes, and as such our main finding that probiotics reduced the counts of live gut bacteria in the blood is considered to be highly significant. Although in our study total counts of bacteria in the blood were decreased by probiotic, none of the single bacteria were not decreased. The reasons for that remain unknown. However, some bacteria translocated to the blood, but some ones never did, indicating the presence of selectivity of intestinal barrier function to bacterial translocation. Regarding the validity of the methodology for analysing gut microbiota in fecal samples, we previously used YIF-SCAN^®^ to show that five potential gut pathogens were approximately 10,000 times less prevalent than six predominant anaerobic groups^24^. For detection of such small numbers of pathogens at subdominant levels, the highly sensitive YIF-SCAN^®^ approach might be appropriate rather than routine DNA-based PCR or next-generation sequencing methods. In particular, YIF-SCAN^®^ for blood microbiota analysis (sensitivity: approximately 1 cell/1 mL-blood)^25^ could lead to the precise analysis of bacterial translocation to the blood. In addition, a previous report using our same method showed a significant correlation between the positivity of bacteria in the mesenteric lymph nodes and blood samples after pancreatoduodenectomy^26^. Therefore, our method in this study can evaluate bacterial translocation.

One previous study showed that individuals with metabolic syndrome had higher concentration of fecal Zonulin and calprotectin and increased *Bacteroidetes*/*Firmicutes* ratio in fecal samples, and that probiotic LcS did not affect this^18^. However, the number of probiotic cells was half of that used in our study (4 × 10^10^ cells), and the study period of 12 weeks was shorter than that in our study (16 weeks). Indeed, in our study bacterial translocation was not reduced at 8 weeks after probiotic administration. Therefore, in order to accurately detect a reduction in bacterial translocation, more probiotic cells and longer administration periods might be necessary.

Regarding the analyses of the gut microbiota in feces in this study, the counts of the *C. coccoides* group and *C. leptum* subgroup were significantly increased at 16 weeks after probiotic administration compared to control, and those of total *Lactobacillus* were increased at 8 and 16 weeks. Additionally, the fecal counts of *L. reuteri* and *L. gasseri* species were significantly increased at 16 weeks after probiotic administration compared to baseline. The *C. coccoides* group of bacteria is known to be one of the most predominant in the human gut^27,28^. According to our previous study^11^, fecal counts of the *C. coccoides* group in type 2 diabetes patients were significantly lower than in patients without diabetes (diabetes/non-diabetes: 9.4 ± 0.8 / 9.8 ± 0.5 log_10_ cells/g of feces). However, in the present study, the fecal counts of the *C. coccoides* group at 16 weeks after probiotic administration recovered to non-diabetes levels (9.8 ± 0.4 log_10_ cells/g of feces), as shown in Table 2. Therefore, some key species in the *C. coccoides* group might be sensitive to probiotics, and a comprehensive analysis is needed to identify which specific bacteria contribute to maintaining gut health in diabetes patients.

Importantly, *L. casei*, *L. reuteri*, and *L. gasseri*, which were present in higher numbers in fecal samples in the probiotic group in this study compared to the baseline. LcS is reported to suppress colon inflammation and play some roles in the maintenance of intestinal barrier function^29^, and reduce LBP levels in diet-induced obesity mice^9^. Especially, counts of *L. casei* in the feces greatly increased after probiotic administration, suggesting the main contribution of LcS to decreased bacterial translocation. Moreover, *L. reuteri* increases mucus thickness in addition to its beneficial effects on the expression of tight junction proteins^30–32^, and *L. gasseri* reduces apoptosis, which could be relevant in protecting epithelial barrier integrity^33^. These two bacteria might, at least in part, contribute to decreased bacterial translocation. However, inflammatory markers such as LBP, IL-6, TNF-α and hs-CRP were not reduced by probiotic administration in our study, and glycaemic control represented by HbA1c, glycoalbumin and FPG did not show clinically significant changes. In previous studies by our group, LcS (4 × 10^10^ cells) administration increased the levels of fecal organic acids and the fecal counts of *Bifidobacterium* in healthy children^34^ and the elderly^35^. Thus, in cases where the intestinal environment is improved by LcS, increased levels of *Bifidobacterium* and organic acids in feces could be expected in addition to the increased levels of *Lactobacillus*. However, this was not found in the present study, suggesting that the effects of LcS on the gut microbiota and intestinal environment might differ between healthy patients and those with type 2 diabetes. Therefore, LcS is considered to only partially improve gut dysbiosis in type 2 diabetes, and further research exploring more efficient probiotics (e.g., multispecies probiotics and/or more probiotic cells) or synbiotics, defined as “mixtures of probiotics and prebiotics”, is necessary.

Our study has several limitations. First, the number of patients (*n* = 68) was too small to evaluate changes of the detection rate of gut bacteria in the blood before and after probiotic administration. Second, as our study did not use a double-blind design, patients were aware they were taking probiotics and may have been familiar with their effects on the gut microbiota, which might have biased the results of the study. Specifically, life style of food habits in the probiotic group might be affected because of a non-blinded study. Third, detection rates of LcS in the feces of the probiotic group were not 100% at 8 and 16 weeks despite instruction of sure consumption of probiotic. However, as its adherence rate was not so low, the influence of adherence on our results was small.

In conclusion, our results showed that probiotic administration reduced bacterial translocation with a partial change of gut microbiota in Japanese patients with type 2 diabetes mellitus. However, in order to further ameliorate gut dysbiosis and reduce chronic inflammation, more efficient procedures may be necessary, including the application of certain synbiotics and/or more probiotic cells with longer administration periods.

## Subjects and Methods

### Participants

Type 2 diabetes patients with stable glycemic control were recruited from the outpatient clinic of Juntendo University Hospital between February 2015 and February 2016. The following inclusion criteria were applied at study registration: 1) 30 < age < 79 years, 2) 6.0 ≤ HbA1c (NGSP)  < 8.0% and 3) treatment with only diet and exercise or medicines excluding α-glucosidase inhibitors. Patients with HbA1c ≥ 8.0% were excluded because their medications might be changed during the study period. The selected patients were excluded from the study if any of the following conditions was diagnosed at registration: 1) serious kidney disease (serum creatinine level ≥ 2.0 mg/dL and / or haemodialysis), 2) serious liver disease excluding fatty liver, 3) inflammatory bowel disease, 4) 20 < body mass index ≤ 35, 5) past history of digestive surgery and 6) not suitable for the study (patients with irregular visits to the hospital and poor adherence to therapy). The study protocol was approved by the Human Ethics Committee of Juntendo University in compliance with the Declaration of Helsinki and current legal regulations in Japan. Written informed consent was obtained from each patient before enrolment in the study. This study was registered on the University Hospital Medical Information Network Clinical Trials Registry, which is a non-profit organization in Japan and meets the requirements of the International Committee of Medical Journal Editors (UMIN000018246, registration date: February 20, 2015).

### Study design

Previous reports showed that the administration of LcS improved the intestinal environment in the elderly (one arm: *n* = 10)^13^ and in healthy individuals with soft stools (one arm: *n* = 17)^12^, respectively. However, a pilot study showed no effects of LcS administration on insulin sensitivity or chronic inflammation in metabolic syndrome (one arm: *n* = 15)^36^. In reference to these previous reports, we aimed to register 70 diabetes subjects (35 patients in each group), taking into consideration the possibility of several dropouts. Thus, 70 patients who met the above criteria were assigned randomly to either the probiotic group, which consumed LcS-fermented milk for 16 weeks, or the control group, which did not receive a probiotic intervention. Randomization was performed using a computer-based dynamic allocation system with minimization procedure to balance for age of patients (Soiken, Inc., Osaka, Japan). The primary endpoints were changes of the gut microbiota, detection rates and bacterial blood counts at the end of the study relative to the baseline values. The secondary endpoints were changes from baseline to the end of the study in HbA1c, lipids and adiponectin, as well as in the following inflammatory markers: high-sensitive C-reactive protein (hs-CRP), lipopolysaccharide binding protein (LBP), tumor necrosis factor-α (TNF-α) and interleukin-6 (IL-6). During the study period, medications for diabetes were not changed. Samples for the analysis of gut microbiota in the blood and feces, fecal organic acids and biochemical assays were obtained after overnight fasts at each hospital visit (0, 8 and 16 weeks).

### Ingestion of LcS-fermented milk

The test beverage was LcS-fermented milk (product name: Yakult 400LT, Yakult Honsha Co., Ltd., Tokyo, Japan). The composition of each 80-ml bottle was as follows: energy, 43 kcal; protein, 1.0 g; lipids, 0.1 g; carbohydrates, 9.5 g; and sodium, 18 mg. The number of LcS cells was 4 × 10^10^ or more at the time of ingestion. The participants in the probiotic group consumed one bottle of LcS-fermented milk every day for 16 weeks at breakfast; this was ensured by up to three telephone calls to each patient, as necessary, just before their visit to the hospital (0, 8 and 16 weeks) during the study period. On the other hand, the participants in the control group consumed no LcS-fermented milk. During the study period, all participants were prohibited from consuming any other probiotics or prebiotics. In addition, at 0 week each physician instructed the participants in the probiotic group to restrict their calorie intake by 40 kcal/day considering the additional calories in the LcS-fermented milk, and monitored adverse events at each visit.

### Determination of bacterial count by 16S and 23S rRNA–targeted reverse transcription-quantitative PCR (RT-qPCR) and qPCR

At each hospital visit, patients defecated at their home, and brought with refrigerants to the hospital. After collecting them, feces were stored immediately at −80 degrees (for organic acids analysis) and 4 degrees (for microbiota analysis), respectively. Fecal samples were weighed and then suspended in 9 volumes of RNAlater^®^ (Ambion, Austin, TX). One milliliter of blood was added to 2.0 ml of RNAprotect bacterial reagent (Qiagen, Hilden, Germany) immediately after collection. After incubation at room temperature for 10 min, the fecal samples were stored at −20 °C. Blood samples were stored at −80 °C, and then transported to the Yakult Central Institute. To quantify the bacteria present in the samples, we examined the gut microbiota composition and plasma levels of the gut bacteria by using the 16S and 23S rRNA–targeted RT-qPCR, Yakult Intestinal Flora-SCAN (YIF-SCAN^®^). Three serial dilutions of the extracted RNA sample were used for bacterial rRNA–targeted RT-qPCR^24,25,37,38^, and the threshold cycle values in the linear range of the assay were applied to the standard curve to obtain the corresponding bacterial cell count in each nucleic acid sample. These data were then used to determine the number of bacteria per sample. The specificity of the RT-qPCR assay using group-, genus- or species-specific primers was determined as described previously^24,25,37,38^. For the enumeration of LcS in feces, Propidium monoazide (Biotium, Inc, CA, USA) treatment of fecal samples, the fecal DNA extraction and qPCR analysis was performed by the methods as described previously^27,39,40^. The sequences of the primers are listed in Supplementary Table 1.

### Measurement of organic acids and pH in fecal samples

The concentrations of organic acids and pH in the fecal samples were measured as described previously^38^. Briefly, the fecal sample was homogenised in 4 volumes of 0.15 µM perchloric acid and allowed to stand at 4 °C for 12 h. The suspension was centrifuged at 20,400 × g at 4 °C for 10 min. The resulting supernatant was passed through a filter with a pore size of 0.45 μm (Millipore Japan, Tokyo). The sample was analysed for organic acids using the Waters HPLC system (Waters 432 Conductivity Detector; Waters Co., Milford, MA) and pH in feces was analysed by IQ 150 pH/Thermometer (IQ Scientific Instruments, Inc., Carlsbad, CA).

In addition, all analyses, including those of the gut microbiota in feces and blood, were performed blindly.

### Biochemical assays

Serum lipids (total cholesterol [T-CHO], high-density lipoprotein cholesterol [HDL-C], low-density lipoprotein cholesterol [LDL-C] and triglycerides [TG]), fasting blood glucose (FBG) and HbA1c were measured with standard techniques. The plasma levels of hs-CRP, IL-6 and TNF-α were measured by latex nephelometry, chemiluminescent enzyme immunoassay and enzyme-linked immunosorbent assay in a private laboratory (SRL Laboratory, Tokyo), respectively. The plasma level of LBP was measured by human LBP ELISA kit (Hycult Biotech, the Netherlands).

### Statistical analyses

All statistical analyses were performed by a private company (Soiken, Inc., Osaka, Japan) with the SAS software version 9.3 (SAS Institute, Cary, NC). Data are expressed as mean ± standard deviation (for normally distributed data) and median (interquartile range; for data with skewed distribution). Comparisons of the results before and after probiotic administration and between the two groups were analysed using the non-parametric Wilcoxon rank sum test and Mann-Whitney U test, respectively. In addition, the analyses by linear mixed model were performed for comparisons of the values between the two groups. The detection rates of fecal and blood bacteria and fecal organic acids between the two groups were analysed by Fisher’s direct test, and comparison of the detection rate of blood bacteria between baseline and 16 weeks was analysed by McNemar’s test. *P* < 0.05 was considered statistically significant.

## Electronic supplementary material

Supplementary information
