# Supplementary material for: Probiotic reduces bacterial translocation in type 2 diabetes mellitus: A randomised controlled study
Source: Sci Rep. 2017 Sep 21;7:12115. doi: 10.1038/s41598-017-12535-9 (PMC5608749; doi:10.1038/s41598-017-12535-9)
Supplement: Supplementary file 1 — Supplementary information [file 41598_2017_12535_MOESM1_ESM.pdf]

Supplementary File

Probiotic reduces bacterial translocation in type 2 diabetes mellitus: A randomised controlled study.

Junko Sato[1], Akio Kanazawa[1,2], Kosuke Azuma[1], Fuki Ikeda[1], Hiromasa Goto[1], Koji Komiya[1], Rei Kanno[1], Yoshifumi Tamura[1,4], Takashi Asahara [5,6], Takuya Takahashi [5,6], Koji Nomoto [5,6], Yuichiro Yamashiro[5], Hirotaka Watada[1,2,3,4]

1) Department of Metabolism & Endocrinology, 2) Center for Therapeutic Innovations in Diabetes, 3) Center for Identification of Diabetic Therapeutic Targets, 4) Sportology Center, 5) Probiotics Research Laboratory, Juntendo University Graduate School of Medicine, 6) Yakult Central Institute

S1 Table Primers used in this study

Target bacteria*	Primer	Sequence (5' - 3')	Ref
Clostridium coccoides group	g-Ccoc-F	AAATGACGGTACCTGACTAA	6
	g-Ccoc-R	CTTTGAGTTTCATTCTTGCGAA	
Clostridium leptum subgroup	sg-Clept-F	GCACAAGCAGTGGAGT	7
	sg-Clept-R3	CTTCCTCCGTTTTGTCAA	
Bacteroides fragilis group	g-Bfra-F2	AYAGCCTTTCGAAAGRAAGAT	8
	g-Bfra-R	CCAGTATCAACTGCAATTTTA	6
Bifidobacterium	g-Bifid-F	CTCCTGGAAACGGGTGG	6
	g-Bifid-R	GGTGTTCTTCCCGATATCTACA	
Atopobium cluster	g-Atopo-F	GGGTTGAGAGACCGACC	7
	g-Atopo-R	CGGRGCTTCTTCTGCAGG	
Prevotella	g-Prevo-F	CACRGTAAACGATGGATGCC	6
	g-Prevo-R	GGTCGGGTTGCAGACC	
Akkermansia muciniphila	AM1	CAGCACGTGAAGGTGGGGAC	9
	AM2	CCTTGCGGTTGGCTTCAGAT	
Clostridium difficile	Cd-lsu-F	GGGAGCTTCCCATACGGGTTG	4
	Cd-lsu-R	TTGACTGCCTCAATGCTTGGGC	
Clostridium perfringens	s-Clper-F	GGGGGTTTCAACACCTCC	2
	ClPER-R	GCAAGGGATGTCAAGTGT	5
Lactobacillus gasseri subgroup	sg-Lgas-F	GATGCATAGCCGAGTTGAGAGACTGAT	2
	sg-Lgas-R	TAAAGGCCAGTTACTACCTCTATCC	
Lactobacillus brevis	s-Lbre-F	ATTTTGTTTGAAAGGTGGCTTCGG	2
	s-Lbre-R	ACCCTTGAACAGTTACTCTCAAAGG	
Lactobacillus casei subgroup	sg-Lcas-F	ACCGCATGGTTCTTGGC	2
	sg-Lcas-R	CCGACAACAGTTACTCTGCC	
Lactobacillus fermentum	LFer-1	CCTGATTGATTTTGGTCGCCAAC	2
	LFer-2	ACGTATGAACAGTTACTCTCATACGT	
Lactobacillus fructiborans	s-Lfru-F	TGCGCCTAATGATAGTTGA	2
	s-Lfru-R	GATACCGTCGCGACGTGAG	
Lactobacillus plantarum subgroup	sg-Lpla-F	CTCTGGTATTGATTGGTGCTTGCAT	2
	sg-Lpla-R	GTTCGCCACTCACTCAAATGTAAA	
Lactobacillus reuteri subgroup	sg-Lreu-F	GAACGCAYTGGCCCAA	2
	sg-Lreu-R	TCCATTGTGGCCGATCAGT	
Lactobacillus ruminis subgroup	sg-Lrum-F	CACCGAATGCTTGCAYTCACC	2
	sg-Lrum-R	GCCGCGGGTCCATCCAAAA	
Lactobacillus sakei subgroup	sg-Lsak-F	CATAAAACCTAMCACCGCATGG	2
	sg-Lsak-R	TCAGTTACTATCAGATACRTTCTTCTC	
Enterobacteriaceae	En-lsu-3F	TGCCGTAACTTCGGGAGAAGGCA	1
	En-lsu-3'R	TCAAGGACCAGTGTTCAGTGTC	
Enterococcus	g-Encoc-F	ATCAGAGGGGGATAACACTT	2
	g-Encoc-R	ACTCTCATCCTTGTTCTTCTC	
Streptococcus	g-Str-F	AGCTTAGAAGCAGCTATTCATTC	3
	g-Str-R	GGATACACCTTTCGGTCTCTC	
Staphylococcus	g-Staph-F	TTTGGGCTACACACGTGCTACAATGGACAA	2
	g-Staph-R	AACAACTTTATGGGATTTGCWTGA	
Pseudomonas	PSD7F	CAAAACTACTGAGCTAGAGTACG	1
	PSD7R	TAAGATCTCAAGGATCCCAACGGCT	
L.casei strain Shirota$	pLcS-57F	CTCAAAGCCGTGACGGTC	10
	pLcS-597R	ACGTGGTGCTAATAATCCTAGTG	

* Group-, genus- or species specific primer sets were developed by using 16S rDNA sequences, except for Cd-lsu-F/R, En-lsu-3F/3'R, and g-Str-F/R, which targeted 23S rDNA.

$ Strain-specific primers sets for *L. casei* strain Shirota were developed by using the LcS-specific sequences (DDBJ/GenBank/EMBL accession number: AB246299).

References

1. Matsuda K, Tsuji H, Asahara T, Kado Y, Nomoto K. Sensitive quantitative detection of commensal bacteria by rRNA-targeted reverse transcription-PCR. Appl Environ Microbiol 2007;73: 32-39.
2. Matsuda K, Tsuji H, Asahara T, Matsumoto K, Takada T, Nomoto K. Establishment of an analytical system for the human fecal microbiota, based on reverse transcription-quantitative PCR targeting of multicopy rRNA molecules. Appl Environ Microbiol 2009;75: 1961-1969.
3. Sakaguchi S, Saito M, Tsuji H, Asahara T, Takata O, Fujimura J, et al. Bacterial rRNA-targeted reverse transcription-PCR used to identify pathogens responsible for fever with neutropenia. J Clin Microbiol 2010;48: 1624-1628.
4. Matsuda K, Tsuji H, Asahara T, Takahashi T, Kubota H, Nagata S, et al. Sensitive quantification of *Clostridium difficile* cells by reverse transcription-quantitative PCR targeting rRNA molecules. Appl Environ Microbiol 2012;78: 5111-5118.
5. Kikuchi E, Miyamoto Y, Narushima S, Itoh K. Design of species specific primers to identify 13 species of *Clostridium* harbored in human intestinal tracts. Microbiol Immunol 2002;46: 353–358.
6. Matsuki T, Watanabe K, Fujimoto J, Miyamoto Y, Takada T, Matsumoto K, et al. Development of 16S rRNA-gene-targeted group-specific primers for the detection and identification of predominant bacteria in human feces. Appl Environ Microbiol 2002;68: 5445-5451.
7. Matsuki T, Watanabe K, Fujimoto J, Takeda T, Tanaka R. Use of 16S rRNA gene-targeted group-specific primers for real-time PCR analysis of predominant bacteria in human feces. Appl Environ Microbiol 2004;70: 7220-7228.
8. Matsuki T. Development of quantitative PCR detection method with 16S rRNA gene-targeted genus- and species-specific primers for the analysis of human intestinal microflora and its application. Nihon Saikingaku Zasshi 2007;62: 255-261. [Article in Japanese]
9. Derrien M. Mucin utilisation and host interactions of the novel intestinal microbe *Akkermansia muciniphila*. Ph.D. thesis (ISBN 978-90-8504-644-8). Wageningen University, Wageningen, The Netherlands, 2007.
10. Fujimoto J, Matsuki T, Sasamoto M, Tomii Y, Watanabe K. Identification and quantification of *Lactobacillus casei* strain Shirota in human feces with strain-specific primers derived from randomly amplified polymorphic DNA. Int J Food Microbiol 2008;126: 210-215.

S2 Table Groups comparison of fecal microbiota at 8 and 16 weeks by linear mixed model analysis

	8 weeks		16 weeks		
	Control	Probiotic	Control	Probiotic	Differences in least mean square (95% CI)
Total bacteria	10.2 (0.1)	10.3 (0.1)	10.2 (0.1)	10.4 (0.1)	0.15 (-0.09, 0.38)
Obligate anaerobes					
C. coccoides group	9.4 (0.1)	9.6 (0.1)	9.5 (0.1)	9.8 (0.1) *	0.25 (0.02, 0.47) *
C. leptum subgroup	9.5 (0.1)	9.5 (0.1)	9.4 (0.1)	9.7 (0.1) *	0.31 (0.02, 0.59) *
Bacteroides fragilis group	8.6 (0.1)	8.6 (0.1)	8.7 (0.1)	8.8 (0.1)	0.08 (-0.24, 0.39)
Bifidobacterium	9.0 (0.1)	8.8 (0.1)	9.0 (0.1)	8.9 (0.1)	-0.14 (-0.53, 0.26)
Atopobium cluster	9.4 (0.1)	9.3 (0.1)	9.4 (0.1)	9.5 (0.1)	0.03 (-0.19, 0.25)
Prevotella	8.3 (0.3)	8.2 (0.2)	8.7 (0.2)	8.6 (0.2)	-0.19 (-0.72, 0.34)
Akkermansia muciniphila	7.9 (0.2)	7.5 (0.3)	7.8 (0.3)	7.6 (0.3)	-0.14 (-0.88, 0.59)
C. difficle	NA	NA	NA	NA	NA
C. perfringens	3.7 (0.9)	5.8 (0.9)	5.3 (0.6)	4.6 (0.6)	-0.72 (-2.58, 1.14)
Facultative anaerobes					
Total *Lactobacillus*	6.1 (0.2)	7.7 (0.2)**	6.6 (0.2)	7.8 (0.2) **	1.17 (0.66, 1.68) **
L. gasseri subgroup	5.6 (0.2)	5.8 (0.2)	5.9 (0.2)	6.0 (0.2)	0.07 (-0.52, 0.66)
L. brevis	3.8 (0.3)	4.6 (0.5)	4.2 (0.7)	5.5 (2.2)	1.31 (-4.00, 6.61)
L. casei subgroup	4.8 (0.3)	7.4 (0.2) **	5.5 (0.3)	7.5 (0.2) **	1.95 (1.20, 2.69) **
L. fermentum	6.8 (0.3)	6.0 (0.3)	6.9 (0.2)	6.5 (0.2)	-0.40 (-1.03, 0.24)
L. fructivorans	NA	NA	NA	NA	NA
L. plantarum subgroup	4.3 (0.2)	4.3 (0.2)	4.4 (0.3)	4.3 (0.3)	-0.05 (-0.86, 0.76)
L. reuteri subgroup	5.3 (0.2)	5.3 (0.2)	5.5 (0.2)	5.5 (0.2)	-0.02 (-0.53, 0.49)
L. ruminis subgroup	5.8 (0.3)	5.6 (0.3)	5.4 (0.3)	5.9 (0.3)	0.47 (-0.36, 1.30)
L. sakei subgroup	5.3 (0.4)	6.0 (0.9)	4.8 (0.4)	4.0 (0.6)	-0.80 (-2.45, 0.84)
Enterobacteriaceae	6.9 (0.2)	7.0 (0.2)	6.8 (0.2)	7.0 (0.2)	0.19 (-0.35, 0.73)
Enterococcus	6.2 (0.2)	6.3 (0.2)	6.0 (0.2)	6.5 (0.2)	0.53 (-0.15, 1.20)
Streptococcus	8.3 (0.1)	8.3 (0.1)	8.5 (0.2)	8.5 (0.1)	-0.02 (-0.44, 0.41)
Staphylococcus	4.4 (0.1)	4.4 (0.1)	4.5 (0.2)	4.4 (0.1)	-0.11 (-0.52, 0.30)
Aerobes					
Pseudomonas	4.0 (0.7)	4.7 (0.6)	4.0 (0.8)	4.5 (0.5)	0.52 (-2.11, 3.15)
Administration of *L. casei* strain Shirota	NA	7.5 (0.0)	NA	8.1 (0.0)	NA

*$p < 0.05$ vs. Control, **$p < 0.01$ vs. Control. The results are expressed as least mean square (SE) (log10 cells/g of feces). NA: Not available, CI: Confidence interval

S3 Table Groups comparison of fecal organic acids and pH at 8 and 16 weeks by linear mixed model analysis

	8 weeks		16 weeks		
	Control	Probiotic	Control	Probiotic	Differences in least mean square (95% CI)
Total organic acids	95.6 (5.5)	93.7 (5.5)	111.5 (5.7)	97.8 (5.7)	-13.67 (-29.97, 2.63)
Acetic acid	54.9 (3.4)	56.2 (3.4)	61.2 (3.3)	55.0 (3.3)	-6.18 (-15.59, 3.22)
Propionic acid	20.0 (1.4)	20.4 (1.4)	24.2 (1.6)	21.3 (1.6)	-2.92 (-7.39, 1.55)
Butyric acid	13.5 (1.4)	11.5 (1.3)	16.9 (1.7)	13.5 (1.5)	-3.48 (-8.04, 1.09)
Isovaleric acid	4.2 (0.6)	3.6 (0.6)	5.6 (0.8)	4.0 (0.7)	-1.65 (-3.75, 0.45)
Valeric acid	3.1 (0.4)	2.2 (0.3)	5.1 (0.8)	2.9 (0.7) *	-2.26 (-4.34, -0.17) *
Succinic acid	3.3 (1.0)	1.4 (1.0)	2.6 (3.3)	7.3 (3.0)	4.71 (-4.38, 13.81)
Formic acid	1.7 (0.4)	1.2 (0.4)	2.1 (0.4)	1.1 (0.4)	-1.03 (-2.19, 0.13)
Lactic acid	NA	NA	NA	NA	NA
pH	6.7 (0.1)	6.8 (0.1)	6.6 (0.1)	6.7 (0.1)	0.15 (-0.06, 0.36)

*$p < 0.05$ vs. Control, The results are expressed as least mean square (SE) (µmol/g of feces). NA: Not available, CI: Confidence interval

S4 Table Groups comparison of clinical parameters at 8 and 16 weeks by linear mixed model analysis

	8 weeks		16 weeks		
	Control	Probiotic	Control	Probiotic	Differences in least mean square (95% CI)
BMI (kg/m^2)	24.1 (0.1)	24.0 (0.1)	24.1 (0.1)	24.1 (0.1)	0.02 (-0.39, 0.43)
HbA1c (%)	6.9 (0.0)	6.9 (0.0)	6.9 (0.1)	7.0 (0.1)	0.08 (-0.09, 0.25)
Glycoalbumin (%)	17.6 (0.2)	17.7 (0.2)	17.6 (0.2)	17.9 (0.2)	0.32 (-0.37, 1.01)
Fasting blood glucose (mg/dL)	129.8 (2.8)	132.1 (2.8)	136.6 (3.2)	133.5 (3.2)	-3.12 (-12.17, 5.92)
Fasting C-peptide (ng/ml)	1.8 (0.1)	1.9 (0.1)	1.8 (0.1)	1.8 (0.1)	0.03 (-0.17, 0.24)
T-CHO (mg/dL)	190.9 (3.1)	192.3(3.1)	191.4 (2.6)	189.7 (2.6)	-1.70 (-9.10, 5.70)
HDL-C (mg/dL)	57.1 (0.9)	54.6 (0.8) *	55.9 (1.2)	54.8 (1.2)	-1.12 (-4.51, 2.28)
TG (mg/dL)	113.1 (8.8)	119.7 (8.7)	121.1 (11.7)	108.7 (11.5)	-12.41 (-45.11, 20.30)
hs-CRP (mg/dL)	814.7 (1187.6)	2631.8 (1187.6)	1238.4 (526.1)	1551.2 (526.1)	312.78 (-1180.52, 1806.08)
TNF-α (pg/mL)	0.9 (0.1)	1.1 (0.1)	1.0 (0.1)	1.1 (0.1)	0.09 (-0.11, 0.29)
IL-6 (pg/mL)	1.9 (0.2)	1.8 (0.2)	2.0 (0.3)	2.1 (0.3)	0.15 (-0.80, 1.10)
Adiponectin (μg/mL)	9.0 (0.2)	9.3 (0.2)	9.2 (0.4)	9.4 (0.4)	0.23 (-0.96, 1.43)
LBP (μg/mL)	10.5 (0.7)	11.4 (0.7)	10.4 (0.6)	10.3 (0.6)	-0.18 (-1.80, 1.44)

*$p < 0.05$ vs. Control, The results are expressed as least mean square (SE), CI: Confidence interval

See Table 1 for abbreviations.
